# Prp19 Arrests Cell Cycle via Cdc5L in Hepatocellular Carcinoma Cells

**DOI:** 10.3390/ijms18040778

**Published:** 2017-04-07

**Authors:** Renzheng Huang, Ruyi Xue, Di Qu, Jie Yin, Xi-Zhong Shen

**Affiliations:** 1 Department of Gastroenterology, Zhongshan Hospital of Fudan University, Shanghai 200032, China; 14111210006@fudan.edu.cn (R.H.); xue.ruyi@zs-hospital.sh.cn (R.X.); 2 Key Laboratory of Medical Molecular Virology, Shanghai Medical College of Fudan University, Shanghai 200032, China; dqu@shmu.edu.cn; 3 Shanghai Institute of Liver Diseases, Zhongshan Hospital of Fudan University, Shanghai 200032, China

**Keywords:** pre-mRNA processing factor 19, cell division cycle 5-like, cell cycle, translation, repressing the translation of mRNA, lysosome-mediated degradation

## Abstract

Pre-mRNA processing factor 19 (Prp19) is involved in many cellular events including pre-mRNA processing and DNA damage response. Recently, it has been identified as a candidate oncogene in hepatocellular carcinoma (HCC). However, the role of Prp19 in tumor biology is still elusive. Here, we reported that Prp19 arrested cell cycle in HCC cells via regulating G2/M transition. Mechanistic insights revealed that silencing Prp19 inhibited the expression of cell division cycle 5-like (Cdc5L) via repressing the translation of Cdc5L mRNA and facilitating lysosome-mediated degradation of Cdc5L in HCC cells. Furthermore, we found that silencing Prp19 induced cell cycle arrest could be partially resumed by overexpressing Cdc5L. This work implied that Prp19 participated in mitotic progression and thus could be a promising therapeutic target of HCC.

## 1. Introduction

Liver cancer, about 70% to 90% of cases of which are hepatocellular carcinoma (HCC), is the fifth most common cancer in men and the seventh in women, and represents the second leading cause of cancer death among men and the sixth leading cause of cancer death among women worldwide [1]. Although surveillance in high-risk population allows diagnosis of HCC at an early stage, most cases still present at advanced and unresectable stage, and limited therapeutic options are available [2]. The development of HCC is a multistep process driving the progressive transformation of normal hepatocytes into highly malignant derivatives [3]. Among them, cell cycle deregulation is a common feature of human cancer [4]. Therefore, targeting the mitotic stage of the cell cycle may be an effective approach for cancer therapy [5].

The highly conserved Pre-mRNA processing factor 19 (Prp19)-associated complex (Prp19C) is a large multi-faceted protein complex that functions in diverse cellular processes, and novel roles of Prp19C are emerging [6]. Prp19C is a critical component of DNA repair and DNA damage checkpoint complexes, which is linked to deleterious consequences-cancer [7]. Prp19C is mainly composed of Prp19, cell division cycle 5-like (Cdc5L), pleiotropic regulator 1 and breast carcinoma amplified sequence 2 [8,9]. Among them, Prp19 forms a tetramer as a key architectural component for Prp19C stability [10]. Prp19 possesses E3 activity [11,12], and plays diverse roles in pre-mRNA splicing [13] and DNA damage response [14,15]. Despite disorders of these cellular events being linked to tumorigenesis, the potential role of Prp19 in HCC is still elusive. Cdc5L has been reported to play a crucial role in mitotic progression. Overexpression of Cdc5L in mammalian cells causes shorter G2 phase and reduced cell size [16] and knockdown of Cdc5L results in dramatic mitotic arrest [17] and chromosome misalignments [18]. Recently, both Prp19 and Cdc5L have been characterized to be overexpressed in HCC tissues and their overexpression are positively correlated with poor prognosis [17,19]. Considering the importance of Prp19 and Cdc5L within this complex, the relationship between them deserves to be investigated in HCC.

In this study, we demonstrate that the levels of Prp19 and Cdc5L are overexpressed in HCC specimens, and positively correlated with each other. In HCC cells, Prp19 binds with Cdc5L and its downregulation results in reduction of Cdc5L. Mechanistic insights reveal that silencing Prp19 compromises translation activity of Cdc5L and facilitates lysosome-induced degradation of Cdc5L. Furthermore, Prp19 knockdown arrests cell cycle at G2/M stage and targeting Prp19-induced mitotic arrest is partially reversed by overexpressing Cdc5L in HCC cells. Taken together, the above findings indicate that Prp19 takes part in the cell cycle regulation, rendering the potential of Prp19 as a diagnotherapeutic target in HCC.

## 2. Results

### 2.1. The Positive Correlation of Prp19 and Cdc5L in HCC

**Figure 1:**
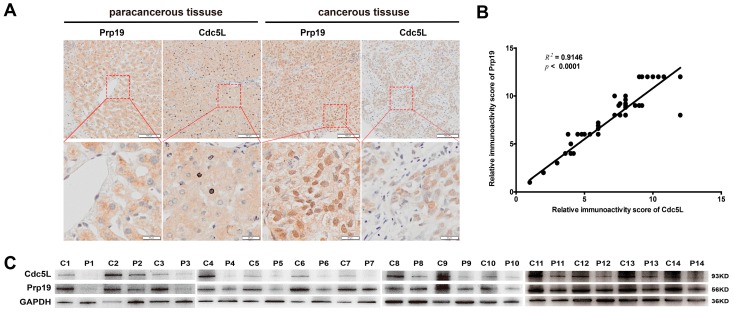
The Positive Correlation of pre-mRNA processing factor 19 (Prp19) and cell division cycle 5-like (Cdc5L) in hepatocellular carcinoma (HCC). (**A**) Paracancerous and Cancerous tissue sections from HCC patients were marked with antibodies against Prp19 and Cdc5L (upper panel, scale bar = 100 μm); Lower panel indicated the amplified view of upper panel (scale bar = 20 μm); (**B**) The positive correlation of Prp19 and Cdc5L immunoactivity score in HCC tissues; (**C**) Prp19 and Cdc5L expression in tumor tissue (C) and paired paratumor tissue (P) from HCC patient specimens using western blot.

To investigate the roles of Prp19 and Cdc5L in HCC tissues, IHC staining was performed with tissues from 69 HCC samples. Our results demonstrated that, in cancerous tissue, Prp19 and Cdc5L were both mainly located at the nucleus, while in paracancerous tissue they were mainly located at the cytoplasm, and this phenomenon was confirmed by confocal laser scanning (Figure S1). In HCC tissues, Prp19 and Cdc5L were more abundant (64/69 for Prp19; 63/69 for Cdc5L) compared to paired paratumor tissue (Figure 1A), which was further confirmed by western blot (13/14) (Figure 1C). Sixty-nine HCC clinical specimens were subjected to analyze the correlation between Prp19 and Cdc5L. Positive correlation between Prp19 level and Cdc5L expression was observed (*R*^2^ = 0.9146, *p* < 0.001; Figure 1B).

### 2.2. Prp19 Binds with Cdc5L and Modulates Cdc5L Expression in HCC Cells

**Figure 2:**
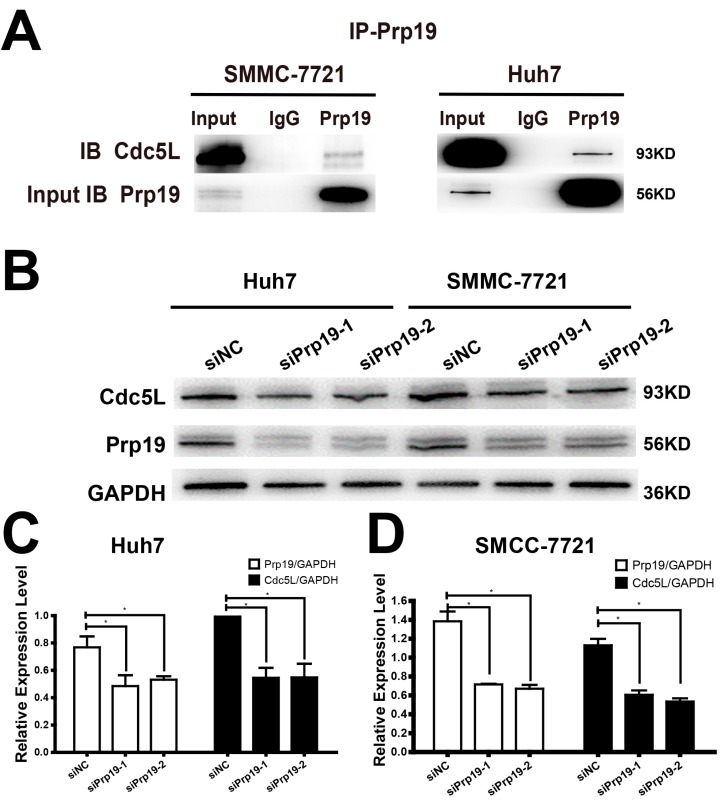
Prp19 binds with Cdc5L and modulates Cdc5L expression in HCC Cells. (**A**) Endogenous interaction between Prp19 and Cdc5L was detected in Huh7 and SMMC-7721 cells; (**B**) Reduction of Cdc5L expression in HCC cells transfected with siRNAs targeting Prp19; (**C**,**D**) The bar charts demonstrate the densitometry ratio of Prp19 or Cdc5L protein to GAPDH (loading control) (* *p* < 0.05). The data are mean ± SD of three independent experiments. NC, control siRNA.

Because of the relationship between Prp19 and Cdc5L described as above, we proposed that Prp19 may infect the expression of Cdc5L. In vivo binding between Prp19 and Cdc5L was observed in Huh7 and SMCC-7721 cells. In contrast to normal mouse IgG, Cdc5L could be detected in the proteins precipitated by anti-Prp19 (Figure 2A), and a similar phenomenon could be detected in another HCC cell line HCCLM3 and HEK293T cell line (Figure S2). Huh7 and SMCC-7721 cells were transfected with siPrp19-1, siPrp19-2 or control siRNA for 48 h. Contrary to the cells transfected with control siRNA, silencing Prp19 obviously inhibited Cdc5L expression in both HCC cell lines (Figure 2B–D).

### 2.3. Prp19 Modulates Cdc5L Expression Via Inhibiting mRNA Translation and Facilitating Lysosome-Mediated Degradation in HCC Cells

**Figure 3:**
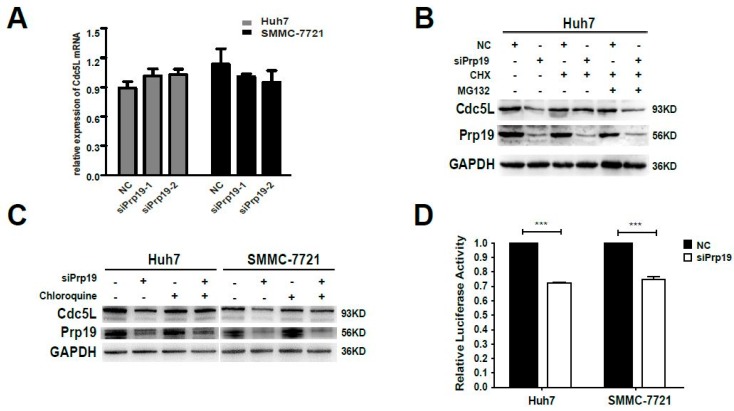
Prp19 modulates Cdc5L expression via inhibiting mRNA translation and facilitating lysosome-mediated degradation in HCC cells. (**A**) The mRNA levels of Cdc5L were measured by qPCR in HCC cells treated with siPrp19 or control siRNA; (**B**) Huh7 cells were transfected with indicated siRNAs. After 48 h transfection, Huh7 cells were treated sequentially with 100 µg/mL CHX for 1 h, 20 µM MG132 for another 6 h, and followed by western blot; (**C**) Huh7 and SMMC-7721 cells were transfected with siPrp19. After 48 h transfection, these cells were treated with 25 µM Chloroquine for another 12 h, and followed by western blot; (**D**) Huh7 and SMMC-7721 cells were transfected with indicated siRNAs. After 24 h transfection, they were co-transfected with control reporter pRL-TK vector and pGL3-5′UTR-Cdc5L for another 48 h, and then luciferase activity was measured (*** *p* < 0.001). Transfections were performed in triplicate, and mean ± SD were calculated for each condition. NC, control siRNA. CHX, cycloheximide.

The decreased expression of Cdc5L protein in siRNA mediated Prp19 knockdown in HCC cells was due to either decreased synthesis or increased degradation. Firstly, qPCR was performed. Inhibiting Prp19 had no significant effect on mRNA level of Cdc5L compared with control siRNA (Figure 3A). Then the post-transcriptional modulation needs to be investigated. Huh7 cells were transfected with indicated siPrp19 or control siRNA for 48 h, and then cultured with cyclohexamide in the absence or presence of the proteasome inhibitor MG132. Endogenous expression of Cdc5L was elevated by treating with cyclohexamide, but the inhibition of the proteasome with MG132 does not prevent the loss of Cdc5L, indicating that proteasome-mediated degradation was not responsible for Prp19-induced modulation of Cdc5L (Figure 3B). Furthermore, we analyzed whether lysosome-mediated degradation worked in this setting. Compared with cells transfected with control siRNA, a decrease of Cdc5L in HCC cells treated with siPrp19 was partially reversed by lysosome inhibitor chloroquine. This suggested that Prp19 facilitated lysosomal degradation of Cdc5L in HCC cells (Figure 3C). Since the translational activity of 5′-UTR is extremely important for growth factors controlling cell cycle [20], the effect of Prp19 on the translational activity of 5′-UTR of Cdc5L in HCC cells was also detected by luciferase activity analysis. We found that the luciferase activity of cells treated with siPrp19 was decreased by about 30% compared to that of cells treated with control siRNA (Figure 3D), indicating that Prp19 modulated the translational activity of Cdc5L. These findings revealed that Prp19 knockdown inhibited Cdc5L expression via facilitating lysosome-mediated degradation and inhibiting mRNA translation in HCC cells.

### 2.4. Prp19 Regulates Mitotic Progression via Cdc5L in HCC Cells

**Figure 4:**
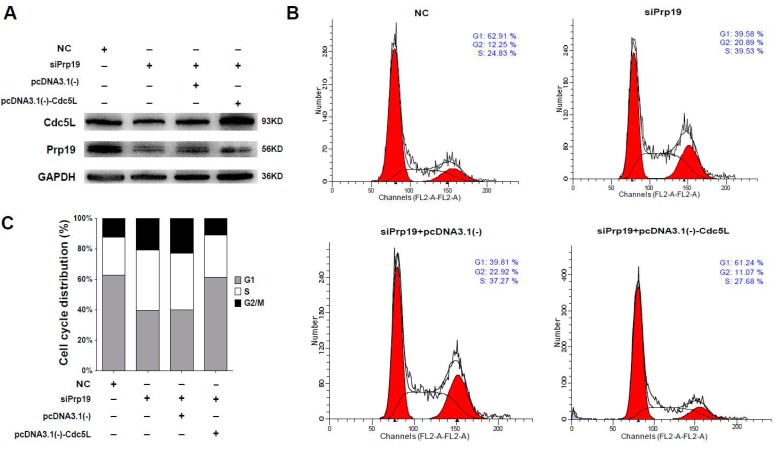
Prp19 regulates mitotic progression via Cdc5L in HCC cells. (**A**) Huh7 cells were transfected with indicated siRNAs. After 24 h transfection, these cells were transfected with indicated plasmids for another 48 h, and followed by western blot; (**B**) Flow cytometry analysis of cell cycle distribution of cells transfected with indicated siRNAs and plasmids in (**A**); (**C**) Quantification of cell cycle shown in (**B**). Data were representative of three independent experiments. NC, control siRNA.

Considering that Cdc5L is a cell cycle regulator of the G2/M transition, we wondered whether Prp19 arrested the cell cycle via Cdc5L. Flow cytometric analysis using cells transfected with targeting siRNAs and plasmids was performed. In Huh7 cells, siRNA targeting Prp19 efficiently inhibited the expression of the Prp19 and Cdc5L, and Cdc5L expressing vector remarkably rescued Cdc5L expression (Figure 4A). In contrast to cells treated with control siRNA, the percentage of G2/M phase increased from 12.25% to 20.89% in the presence of siPrp19. Prp19 knockdown-induced elevation of the percentage of G2/M phase was inhibited by exogenous expression of Cdc5L (decreased from 22.92% to 11.07%) (Figure 4B,C). A similar phenomenon was observed in SMMC-7721 cells (Figure S3). These results indicated that Prp19 participated in mitotic progression via Cdc5L in HCC cells.

## 3. Discussion

As one of the leading causes of cancer-related deaths in the world, the survival rate of HCC is very low [1,2], which is mainly due to the complicated molecular mechanism of HCC. Lately, aberrant expression of Prp19 was observed in most HCC tissues and was positively correlated with vascular invasion, tumor capsule breakthrough and poor prognosis [19]. Prp19 may inhibit chemotherapeutic drugs-induced apoptosis in HCC cells [15]. These findings indicate the important role of Prp19 in the development of HCC. Cdc5L, another core component of the Prp19C, is found to modulate the expression of a set of genes involved in the mitosis and facilitate mitotic processing [21]. In an in-depth study, it was shown that Prp19 exerts bioactivities alone or as a part of a protein complex in tumor formation [22]. This study demonstrated the positive correlation of Prp19 and Cdc5L in HCC tissues and identified the interaction between them in HCC cells. Considering that Cdc5L is involved in cell cycle processing, we also found that Prp19 facilitated G2/M transition in HCC cells. Furthermore, Prp19 knockdown-induced cell cycle arrest was partially reserved by overexpression of Cdc5L.

Recently, Mu et al. reported the mutual modulation of components within Prp19C and hypothesized that this effect might be due to the requirement for each component to maintain complex stability [21]. Our study firstly proved that Prp19 modulated Cdc5L expression via inhibiting mRNA translation and facilitating lysosome-mediated degradation in HCC cells, which further verified the hypothesis proposed elsewhere [21]. It has been reported that the Trp-Asp forty-amino-acid repeat (WD-40 repeat) domain of Prp19 is essential for protein binding [23] and Prp19 forms tetramers to provide a scaffold for Prp19C organization [10]; therefore, Prp19 may provide an interaction platform to maintain complex stability and avoid lysosome-mediated degradation. Depletion of Prp19 may impair mRNA translation of components within this complex.

This study firstly revealed that both Prp19 and Cdc5L were overexpressed and positively correlated in HCC tissues. Furthermore Prp19 bound with Cdc5L and participated in mitotic processing by modulating Cdc5L. Recently, therapeutic modalities such as anti-mitotic drugs are booming [4], and some pharmaceutical products exert antitumor effects by inducing cell cycle arrest at the G2/M phase [24,25,26],suggesting that the inhibition of Prp19C could be a targeted anti-mitotic cancer therapy. This may provide theoretical basis to elucidate the role of Prp19C during the pathogenesis of HCC.

## 4. Materials and Methods

### 4.1. Patients and Samples

All 69 HCC samples were randomly retrieved from patients who underwent curative resection and verified by post-operational histopathology at Liver Cancer Institute, Zhongshan Hospital of Fudan University from 1 December 2006 to 31 December 2008. The clinic pathologic characteristics of 69 patients were summarized in Table S1. The protocol was approved by the Institutional Ethics Committee of Zhongshan Hospital of Fudan University (Shanghai, China) at 25 February 2016 (Code, Y2016-042), and written informed consent was obtained from all study participants.

### 4.2. Immunochemistry and Immunofluorescence

Slides of paraffin-embedded primary HCC tissues were used for immunochemistry and stained with antibodies against Prp19 and Cdc5L. The exact procedure and the semi-quantitative method were reported elsewhere [27]. Images were processed with a Nikon microscope with NIS Element F3.2 software (Nikon, Melville, NY, USA).

When subjected to immunofluorescence assay, unfixed frozen sections were generally processed as described below, briefly. First, sections were incubated with rabbit anti-human Prp19 polyclonal antibody (1:250) and mouse anti-human Cdc5L Monoclonal antibody (1:20) overnight at 4 °C after blocked by Phosphate buffered saline (PBS) containing 5% Bovine serum albumin (BSA). They were then washed by PBS carefully. These sections were incubated with Alexa Fluor^®^ 647-conjugated AffiniPure Donkey Anti-Rabbit IgG and Alexa Fluor^®^ 488-conjugated AffiniPure Donkey Anti-Mouse IgG (both 1:500) at room temperature for 2 h (Jackson ImmunoResearch Inc., West Grove, PA, USA). Finally, sections were added with Mounting Medium for Flurescence with DAPI and detected by Leica TCS SP5 confocal microscope (Leica Microsystems, Wetzlar, Germany).

### 4.3. Cell Lines, Plasmids, siRNAs, Reagents and Chemical Agents

Huh7, SMMC-7721, HCCLM3 and HEK293T cells were obtained from the Cell Bank of the Chinese Academy of Sciences (Shanghai, China) where they were characterized by mycoplasma detection, DNA-Fingerprinting, isozyme detection and cell vitality detection. All cell lines were cultured in DMEM with 10% fetal bovine serum. All these cells were cultured in a humidified incubator at 37 °C in the presence of 5% CO_2_. Human Cdc5L in pcDNA3.1/myc-His(−) vector was generated according to the standard PCR protocol. All primers and siRNAs sequences were listed in Table S2. Transfection of plasmids and siRNAs were performed with Lipofectamine^®^ 2000 transfection reagent (Invitrogen, Carlsbad, CA, USA) following the manufacturer’s protocol. Antibodies recognizing Prp19 and Cdc5L were purchased from Abcam Ltd. (Cambridge, UK). Antibodies against GAPDH was from Cell Signaling Technology (Beverly, MA, USA). Chloroquine was dissolved in sterile nuclease-free water (Cell Signaling Technology, Beverly, MA, USA). Cycloheximide was dissolved in sterile nuclease-free water and used at 100 µg/mL (Sigma-Aldrich, St. Louis, MO, USA). MG132 was dissolved in dimethyl sulfoxide (DMSO) and used at 20 µM (Merck KGaA, Darmstadt, Germany).

### 4.4. Real-Time PCR (qPCR)

Total RNA was exacted by TRIzol (Invitrogen, Carlsbad, NY, USA). The first strand cDNA synthesis was carried out with AMV RNA PCR kit (TaKaRa, Dalian, China) according to the manufacturer’s protocol. Subsequent qPCR was performed using a SYBR Green Premix Ex Taq (TaKaRa) on ABI StepOne Plus system (Applied Biosystems, Foster City, CA, USA). The relative mRNA level of specific genes was calculated using the dd*C*_t_ method after normalization against GAPDH. The primers of interested genes were as follows:

Prp19: 5′-GTGCC AAGTT CCCAA CCAAG TGTT-3′ (forward) and 5′-AGCAC AGTGG CTTTG TCTTG AAGC-3′ (reverse);

GAPDH: 5′-TCGAC AGTCA GCCGC ATCTT CTTT-3′ (forward) and 5′-GCCCA ATACG ACCAA ATCCG TTGA-3′ (reverse);

Cdc5L: 5′-TCTCT GAAG CTCC TCTC GGC-3′ (forward) and 5′-CATC CTCG GTAT TCCT CCAT ACG-3′ (reverse).

### 4.5. Western Blot Assay

For western blot, cells were lysed in lysis buffer (20 mM Tris-HCl, pH 7.5, 150 mM NaCl, 1 mM EDTA, and 1% Triton X-100 with protease inhibitor), and their extracts were clarified via centrifugation. The cell lysate proteins were separated on 10% SDS-PAGE gel and then transferred to polyvinylidene difluoride membrane (Millipore, Billerica, MA, USA). The membranes were blocked in blocking solution (50 mM Tris-HCl, 150 mM NaCl, 5% (*w*/*v*) non-fat dry milk and 0.1% Tween-20) at room temperature for 2 h, followed by incubation with appropriate primary antibodies at 4 °C overnight. After incubating with appropriate secondary antibodies at room temperature for 1 h, gels were scanned and quantified using ImageQuant LAS 4000 mini (GE Healthcare, New York, NY, USA).

### 4.6. Immunoprecipitation Analysis

Briefly, Huh7 and SMCC-7721 cells under corresponding treatments were harvested in immunoprecipitation lysis buffer supplemented with a complete protease inhibitor cocktail and a phosphatase inhibitor cocktail (Roche, Mannheim, Germany). The cell lysates were immunoprecipitated with corresponding antibodies and detected by western blot.

### 4.7. Dual Luciferase Analysis

First, pGL3-5′UTR-Cdc5L, which contains a 299 bp promoter fragment of mRNA of Cdc5L inserted upstream of firefly luciferase coding sequences, was constructed from pGL3-Basic Vector, which lacks eukaryotic promoter and enhancer sequences and allows inserted upstream of the promoter. After the treatment of siPrp19 or control siRNA, Huh7 and SMCC-7721 cells were co-transfected with control reporter pRL-TK Vector and pGL3-5′UTR-Cdc5L. Luciferase activities were measured 48 h after transfection. For each condition, the ratio of firefly to Renilla luciferase was determined and normalized to the value obtained for HCC cells transfected with control siRNA (Relative Expression). The value of the sample transfected with control siRNA was set at 100%. Data are presented as the means ± SEM of three independent experiments (in triplicate).

### 4.8. Flow Cytometric Analysis

For flow cytometric analysis, cells were collected in phosphate-buffered saline (PBS) containing 2 mmol/L ethylenediaminetetraacetic acid and fixed in cold ethanol (70–80%) for at least 18 h. Fixed cells were washed three times in PBS and then permeabilized with 1% TritonX-100 for 20 min and incubated with 1 mg/mL RNase A in PBS for 20 min. Subsequently, the cells were stained with 50 µg/mL propidium iodide (PI) (Becton–Dickinson, San Jose, CA, USA) in PBS-TritonX-100 for an additional 20 min at 4 °C. At last, the cells were analyzed for cell cycle distribution using a BD FACSCalibur (Beckton Dickinson, Franklin Lakes, NJ, USA) flow cytometer and analyzed using Cell Quest software supplied by the manufacturer.

### 4.9. Statistical Analysis

Statistical analyses were conducted on SPSS 19.0 for Windows. Student’s *t*-test was used to compare differences between different groups (* *p* < 0.05, ** *p* < 0.01 and *** *p* < 0.001).

## Supplementary Materials

Supplementary materials can be found at www.mdpi.com/1422-0067/18/4/778/s1.
