# Supplementary material for: Prp19 Arrests Cell Cycle via Cdc5L in Hepatocellular Carcinoma Cells
Source: Int J Mol Sci. 2017 Apr 7;18(4):778. doi: 10.3390/ijms18040778 (PMC5412362; doi:10.3390/ijms18040778)
Supplement: Supplementary file 1 [file ijms-18-00778-s001.pdf]

Supplementary Materials: Prp19 Arrests Cell Cycle via Cdc5L in Hepatocellular Carcinoma Cells

Renzheng Huang, Ruyi Xue, Di Qu, Jie Yin and Xi-Zhong Shen

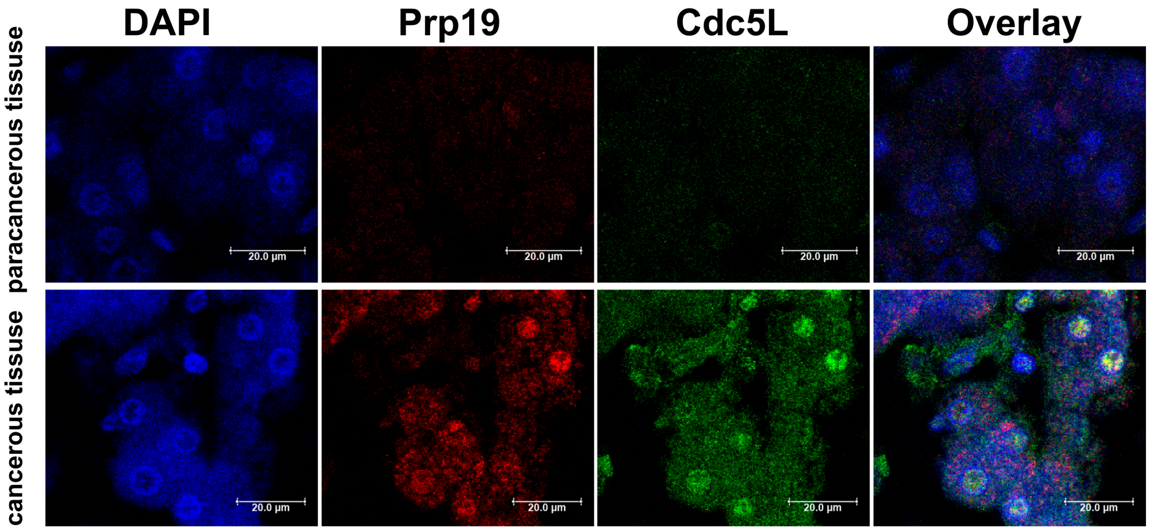

Figure S1. Immunofluorescence microscope images of Prp19 and Cdc5L in HCC and non-HCC tissue. (Upper panel, non-HCC tissue; Lower panel, HCC tissue).

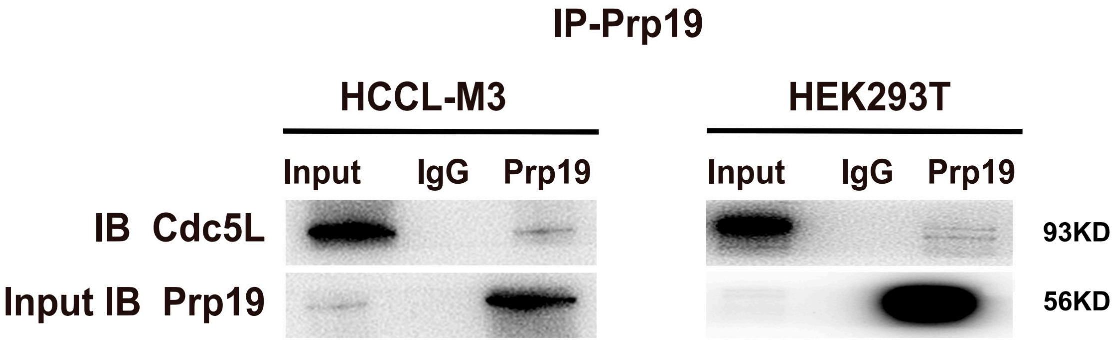

Figure S2. Endogenous interaction between Prp19 and Cdc5L was detected in HCCLM3 and HEK293T cells.

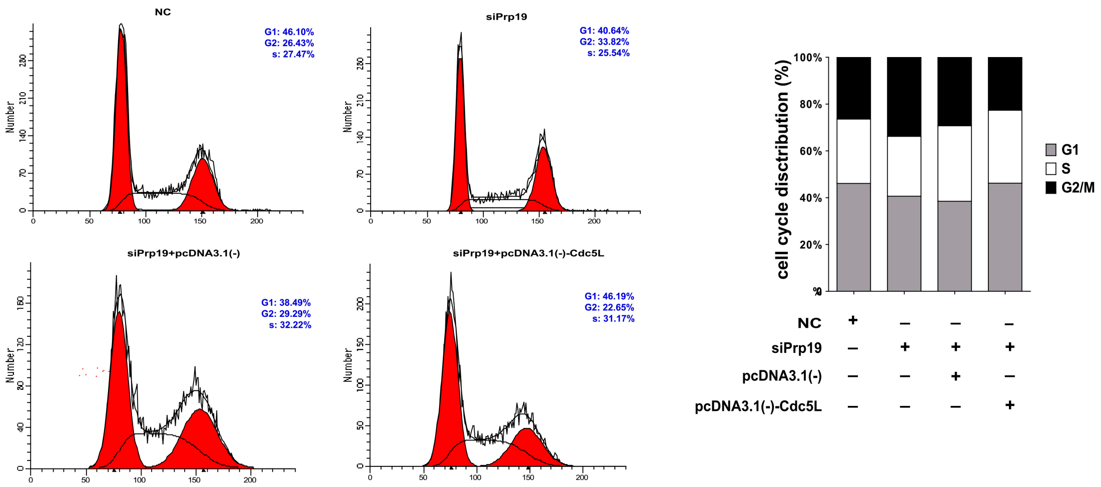

Figure S3. Flow cytometry analysis of cell cycle distribution of SMMC-7721 cells transfected with indicated siRNAs and plasmids in Figure 4A. Quantification of cell cycle shown in right panel.

Table S1. Clinic pathological features in relation to the Cdc5L and Prp19 expression of HCC tissues

Characteristics		Total	Cdc5L		Prp19	
			Low	High	Low	High
Gender	Female	10	0	10	0	10
	Male	59	22	37	21	38
Age (years)	<50	20	4	16	4	16
	≥50	49	18	31	17	32
HbsAg	Negative	16	7	9	7	9
	Positive	53	15	38	14	39
AFP (ng/mL)	<400	38	13	25	13	25
	>=400	31	9	22	8	23
Cirrhosis	Negative	12	6	6	6	6
	Positive	57	6	51	5	52
Tumor size (cm)	<5	45	14	31	12	33
	≥5	24	8	16	9	15
Tumor number	Single	47	14	33	13	34
	Multiple	22	8	14	8	14
AJCC stage	I and II	49	16	33	15	34
	III and IV	20	6	14	6	14

Table S2. List of siRNAs for suppressing Prp19 and control siRNA

siRNAs	Sequence (5′ to 3′)
siRNA1 Prp19 sense	GCC ACU AUC AGG AUU UGG UTT
siRNA1 Prp19 antisense	ACC AAA UCC UGA UAG UGG CTT
siRNA2 Prp19 sense	CUU GAA GGA ACG UAC UAA UTT
siRNA2 Prp19 antisense	AUU AGU ACG UUC CUU CAA GTT
siRNA Negative control sense	UUC UCC GAA CGU GUC ACG UTT
siRNA Negative control antisense	ACG UGA CAC GUU CGG AGA ATT
